# Colorectal Cancer Screening Prevalence, Perceived Barriers, and Preference for Screening Colonoscopy Among Hospitalized Women

**DOI:** 10.5152/tjg.2022.21567

**Published:** 2022-11-01

**Authors:** Howard Graham, Regina Kauffman, Waseem Khaliq

**Affiliations:** 1 Division of Hospital Medicine, Department of Medicine, Johns Hopkins Bayview Medical Center, Johns Hopkins University School of Medicine, Baltimore, Maryland, USA

**Keywords:** Colorectal cancer, hospitalized women, non-adherent women, screening colonoscopy

## Abstract

**Background:**

Barriers to colorectal cancer screening persist despite screening campaigns, especially among women. This study explores the prevalence, preferences, and barriers associated with colorectal cancer screening and evaluates the effect of an inpatient intervention (one-on-one bedside education and handout about colorectal cancer) on screening adherence among hospitalized women.

**Methods:**

A prospective intervention study among 510 hospitalized women, who are cancer-free (except for skin cancer) at enrollment, aged between 50 and 75 years was conducted at an academic center. Socio-demographic, family history, and medical comorbidities data were collected for all patients. A post-hospitalization follow-up survey determined the effect of inpatient intervention on colorectal cancer screening adherence. Unpaired *t*-test and chi-square tests were used to compare characteristics, perspectives, and preferences for screening among adherent and non-adherent groups.

**Results:**

Mean age was 60.5 years, 45% reported an annual household income of <$20 000, 36% of women were African American, 27% of women were overdue for colorectal cancer screening, and 33% never had a screening colonoscopy. The most frequently reported barriers to colorectal cancer screening were “I have other problems more important than getting a colonoscopy,” “No transportation to get to the test,” and “Not counseled by primary care provider.” Sixty-six percent of the non-adherent women would agree to have an inpatient screening colonoscopy if offered.

**Conclusion:**

A significant number of hospitalized women are non-adherent to colorectal cancer screening, while the educational intervention was partially successful in enhancing colorectal cancer screening, most hospitalized women remained non-adherent after hospitalization. A majority of these women were amenable to inpatient screening colonoscopy if offered during a hospital stay.

**Main Points**

- A significant number of hospitalized women are non-adherent to colorectal cancer screening guidelines.
- Lack of transportation and not being counseled by primary care are among the major barriers to screening. A majority (66%) of non-adherent women are amenable to inpatient screening colonoscopy if offered during a hospital stay.
- Offering inpatient colorectal cancer screening to non-adherent patients may lower the burden of cancer screening disparities among women, especially those who are at high risk.

## Introduction

Colorectal cancer (CRC) is the third most commonly diagnosed non-skin cancer (~8% of newly diagnosed cancers) and the third most common cause (~9% of all cancers death) of cancer deaths among women in the United States.^1^ In 2021, an estimated 51 680 new cases of colon cancer and 18 300 cases of rectal cancer are expected to be diagnosed in women and approximately 24 460 women are expected to die from colon cancer in the United States.^1^ Colonoscopy every 10 years is among the most frequently used test for CRC screening and among the most effective in reducing CRC mortality in the United States.^2^ Colorectal cancer incidence and mortality rates have been declining for the past 2 decades among adults aged 50 years and older, which is largely attributable to prompt screening and early detection.^3^

Approximately, 4.1% of women (1 in 25) will be diagnosed with CRC in their lifetime.^4^ A US population-based survey indicates that current CRC screening rates among women aged 50-75 years are 63.4% (95% CI, 61.7-65.0). Although CRC screening utilization rates among women significantly increased (~29%) from 2000 to 2010, the disparity in screening utilization persists by age (younger age group, 50-64 years), education, income, race, access to health care, and insurance.^5^ The race disparity was more pronounced among non-Hispanic Asian followed by Hispanic women, 54.7% and 59.3%, respectively.^5^ The CRC screening is especially lowest among women with low socioeconomic status and those with a lack of access to healthcare (uninsured women 45.4% and with no usual source of care 45.9%).^5-9^ Despite proven mortality benefits, about one-third of eligible adults in the United States have never been screened for CRC, and offering choices in CRC screening strategies may increase screening uptake.^10,11^ Adherence to CRC screening remains low among women in general as compared to men.^9^ This gender disparity persists even after stratification for income, education, and access to health insurance coverage as reported by a systematic review.^9^ Thus, it becomes essential to evaluate perspective, perception, and preference for CRC screening among women to better understand gender-related barriers as women may perceive the risk of developing CRC differently than men.

The purpose of the current study was to evaluate the prevalence of non-adherence to CRC screening among hospitalized women and to determine their perspective, barriers, and preference for CRC screening. The study also evaluated whether an inpatient intervention that included one-on-one bedside education about CRC screening would improve their adherence to screening. We hypothesized that inpatient screening education would increase CRC awareness and hence improve adherence to CRC screening.

## Materials and Methods

### Study Design and Sample

The Institutional Review Board at the academic center approved the study proto­col. All study participants provide their written informed consent for study participation.

In this prospective intervention study, all women between 50 and 75 years of age and admitted to the general medical service at an academic center were approached for study participation from December 1, 2014, to May 31, 2017. In addition to age and gender, the other major eligibility criteria were to enroll women who were cancer-free (except for skin cancer) at baseline. Patients who were hospitalized with an admission diagnosis of acute myocardial infarction, acute confusion or change in mental status, acute pulmonary embolism, acute cerebrovascular event, acute fulminant hepatitis, or pregnancy were excluded from the study participation. In addition to the above-mentioned, patients with serious comorbidities with a life expectancy of less than 10 years like dementia, end-stage liver disease, acquired immunodeficiency syndrome, hospice care, and end-stage renal disease on hemodialysis were also excluded. Patients who had multiple admissions during the study period were only enrolled on their first visit. A total of 1302 women were eligible by the study criteria for participation during the study period. Of these hospitalized women, 423 (32%) refused to participate and 367 (28%) women were discharged from the hospital before the study coordinator could consent them. Two patients dropped out of the study after agreeing to participate. Thus, the final study consisted of 510 (40%) women.

### Protocol and Measures

A bedside survey was conducted pre-study intervention to collect data consisting of socio-demographic information such as race, marital status, education, and annual household income. Adherence to CRC was self-report and defined according to the United States Preventive Services Task Force recommendations that all individuals aged 50-75 years should undergo CRC screening via stool-based or direct visualization tests for CRC screening.^12^ Thus, women were considered non-adherence to CRC screening if they had a screening Fecal Occult Blood Test (FOBT) more than a year ago, flexible sigmoidoscopy more than 5 years ago, or screening colonoscopy more than 10 years ago.^12^ Several questions regarding CRC risk factors including personal history of Lynch syndrome or familial adenomatous polyposis or inflammatory bowel disease were included. Family history of CRC was judged to be positive in subjects reporting a CRC diagnosis in first-degree relatives (namely father, mother, brothers, sisters, sons, or daughters). We evaluated access to health care with the variables of health insurance status and having a primary care physician. Disease burden was characterized by assessing medical comorbidities including those needed for the Charlson Comorbidity Index (CCI), a method of categorizing comorbidities by mortality risk status based on the International Classification of Diseases diagnosis codes.^13^ Both CCI and comorbidities other than CCI were ascertained from the medical records of the enrolled women. Health behavior in general was assessed by asking about smoking status, alcohol use, and screening for cancers other than CRC. Several Likert scale style questions were asked to the study population to evaluate perceived susceptibility, benefits, and barriers to CRC screening. Prior to the study intervention, we also elicited preferences of these hospitalized women for CRC screening locale provided it can be performed anywhere; post-intervention, we inquired if these women were willing to get a screening colonoscopy post-hospitalization.

### Study Intervention

The study coordinator provided one-on-one bedside education about CRC screening via sharing a handout and counseled about the importance/benefits of screening.^14^ Each participant was given a $10 gift card at the end of this study enrollment survey.

### Post Intervention Measures

Six months after discharge from the hospital, study participants started to receive a follow-up phone survey to determine their adherence status to CRC screening. This short follow-up survey also elicited if patients had followed up with their primary care provider and underwent screening colonoscopy post-hospitalization after the study intervention. Up to 3 attempts were made to reach out to the study participants for follow-up phone survey.

## Outcome and Evaluation

The primary outcome was the prevalence of non-adherence to CRC screening among hospitalized women, including their preferences and perceived barriers to CRC screening. The secondary outcome was to evaluate if the pattern of non-adherence to CRC screening persists or changes over a period after one-on-one bedside educational intervention during hospitalization.

## Statistical Methods

Study participants’ characteristics are presented as proportions and means. Unpaired *t*-test and chi-square tests were used to compare perspective, barriers, and receptivity toward CRC screening between women who were adherent and non-adherent to CRC screening. These tests determined significance at *P* ≤ .05. The data were analyzed using the Stata statistical software (StataCorp LP, Version 13.1).

## Results

The mean age of the population studied was 60.5 years (standard deviation [SD] = 6.9), 45% of the study population reported an annual household income of less than $20 000, 36% were African American, and only 2% of the study population was uninsured (Table 1). Over one-quarter (27%) of the study population was non-adherent to CRC screening guidelines and 33% of the women never had screening colonoscopy. A total of 12% of the study population reported having a first-degree relative with a history of CRC and 9% were at high risk for CRC. Detailed study population characteristics can be seen in Table 1.

Non-adherent women lacked awareness about CRC screening, as these women were less likely to hear/watch advertisements about CRC screening or know the tests for CRC screening (Table 2). There was no difference in perceived susceptibility among the 2 groups except that adherent women were more likely to know anyone who had screening colonoscopy and encouraged by anyone who had screening colonoscopy. Several barriers to CRC screening were perceived differences between the 2 groups, including the most cited barrier to screening: “I have other problems more important than getting a colonoscopy,” “No transportation to get to the test,” “Not counseled by my doctor about the need to do it,” “I am afraid to have a colonoscopy because I don’t understand what will be done,” and “I am afraid to have a colonoscopy because I might find out something is wrong” (Table 2).

Non-adherent women were also less likely to have screening colonoscopy and were also less likely to have been counseled about the importance of CRC screening by their primary care provider (Table 3). Prior to study intervention, only half of the women enrolled in the study would prefer to undergo screening colonoscopy during hospitalization and 8% would not undergo screening at all regardless of the clinical locale of the screening test. Only 6% (n = 32) of women reported that someone had talked to them about CRC screening during their inpatient stay. After the study intervention (bedside one-on-one education with hand out and encouragement from the study coordinator), almost all women (89%) believed that it is important for healthcare providers to discuss CRC screening while patients are in the hospital. More than three-quarters (78%) of the enrolled women indicated that they would agree to have an inpatient screening colonoscopy if it was due, and it was offered. Eighty-three percent of the adherent women and 66% of the non-adherent women (*P* < .001) confirmed that they would be willing to have a screening colonoscopy during the hospital admission (Table 3).

To measure the effect of the study intervention on screening adherence, a follow-up survey was conducted after discharge from the hospital. We were able to reach only 23 (17%) non-adherent women over the phone for a follow-up survey. Of these, only 30% (n = 7) of women reported receiving a screening colonoscopy since the discharge. The mean follow-up period was 26 months (SD = 12), although the follow-up calls were initiated 6 months post-hospitalization. About 87% (n = 20) of these women had a follow-up with their primary care provider since discharge; however, 85% (n = 17) discussed screening colonoscopy with their primary care provider, 79% (n = 15) of women felt that their primary care provider spent enough time discussing cancer screening, and 35% women (n = 8) reported hospital readmission since enrollment with an average of 2.5 (SD = 1.3) hospital admission since study intervention.

## Discussion

Our study found that more than a quarter of hospitalized women were non-adherence to CRC screening guidelines. We also report that one-third of the hospitalized women never had a screening colonoscopy, nearly half of the women were from a low-income group, and one-third were African American. There were no differences in perceived susceptibility between the 2 groups apart from the anticipated painful procedure. This barrier should be conceptualized knowing that a small number (15%) of non-adherent women had undergone a colonoscopy and a majority reported a lack of understanding of the procedure, lack of bowel preparation for screening, and fear of finding something wrong. Although we noted that both adherent and non-adherent women perceived the benefits of CRC screening equally well, reported barriers to screening colonoscopy were significant among the 2 groups. Lack of transportation, having other problems more important than getting CRC screening, and not being counseled by primary care providers stood up among the 3 more frequently cited barriers by non-adherent hospitalized women. Post-study intervention, most women from both adherent and non-adherent groups thought that it was important for health care providers to discuss CRC screening during hospital stay and expressed willingness to undergo screening colonoscopy if it was due and offered during hospitalization. During post-hospitalization follow-up via phone survey (where up to 3 attempts were made to reach out to the non-adherent women), only a small number of women ended up getting a screening colonoscopy, although most of these women did follow up with their primary care provider and discussed the CRC screening during a follow-up visit. Post-hospitalization results should be interpreted with caution, as we were only able to reach approximately 17% of the non-adherent women. Nevertheless, this study provides some insight into the pattern of non-adherence to CRC screening among hospitalized women after discharge from the hospital.

Only a few studies have evaluated the prevalence of non-adherence to CRC screening among the hospitalized population.^15,16^ Results from our study were consistent with prior study, reiterating the fact that a pattern of adherence to CRC screening among hospitalized populations does not change over time.^15^ Perceived risk and knowledge about CRC are thought to influence the perceptions of screening necessity and are crucial to adopting preventive behavior.^17^ Our results are also consistent with previous studies reporting a lack of knowledge as a predominant barrier to CRC screening participation, especially among low-income groups and ethnic minorities.^18-20^ It is interesting to note that fewer non-adherent women reported getting counseled about the importance of CRC screening by their primary care provider. Several studies have reported that women were less likely to receive a recommendation from the physician for CRC screening making non-adherence a physician-related barrier.^9^ This may reflect the lack of knowledge among the hospitalized women about CRC screening not only due to low socioeconomic status and poor access to health care resources but also lack of recommendations by their primary care providers.

Perceived risk of cancer has been seen as a primary motivator in increasing compliance with screening.^21-23^ Although there are conflicting data on whether women view the perceived risk of CRC lower than that of breast or cervical cancer, women who are up to date with their breast and cervical cancer screening are more likely to be compliant with CRC screening.^9,21-24^ Our study also suggests a high rate of non-adherence to screening mammography among women who were non-adherent to CRC screening.

The study intervention provided one-on-one bedside education as a preferred method to improve awareness and address perceived barriers or anxiety associated with the CRC screening tests.^25^ The intervention’s influence on the study population can be evaluated by fact that a majority of women welcomed the idea of healthcare provider counseling CRC screening during hospitalization and would agree to an inpatient screening colonoscopy if due and offered during the hospital stay. This finding is in contrast with a previous study where only one-fifth of the non-adherent hospitalized patients reported a willingness to undergo inpatient colonoscopy.^15^ However, overall findings from our study are consistent with a previous study evaluating preferences of hospitalized women toward other cancer screening as noted for breast cancer.^26^

While the outpatient setting has traditionally been seen as the optimal location for counseling and screening for CRC, lack of awareness about testing and inadequate outpatient provider counseling are factors associated with a low prevalence of screening, particularly in socioeconomically disadvantaged groups.^27-32^ Moreover, some of the identified barriers to CRC screening, like lack of time, lack of transportation, a long waitlist to schedule, have other problems more important than screening, and not having anyone to help the patient during the procedure, can be overcome if screening can be performed during hospitalization.^33^ It has been suggested that multilevel screening strategies are warranted to address the multiple barriers affecting CRC screening adherence.^34^ Hospitalized patients having a serious health event are vulnerable and more receptive to health advice and inspired to seek and incorporate preventative measures for their physical wellbeing.^35^ Offering counseling and CRC screening education to patients whenever and wherever they come into contact with the healthcare system regardless of clinical locale may be one of the ways to capture this vulnerable population.

Currently, inpatient CRC screening is not a common occurrence in the United States;^16^ however, our data suggest that hospitalized women represent a captive audience who are receptive to any recommendations provided by their hospital providers. Providing inpatient CRC screening counseling in the same way as other population health promotions like smoking cessation, diabetes education, influenza, and pneumococcal vaccination may potentially increase CRC screening adherence. Additionally, making CRC screening available to hospitalized women at a time when patients are willing and available to undergo the test could improve adherence in underserved and high-risk populations. In our study’s post-hospitalization follow-up, a majority of respondents reported a follow-up with their primary care physician where cancer screening was adequately discussed. However, this information should be extrapolated with caution due to low follow-up response rate.

Several limitations of this study should be considered. First, this study was conducted at a single academic hospital in an urban setting. Second, our study population consisted of hospitalized women only; however, this should be considered a strength as female gender is reported by several studies to be a barrier to CRC screening adherence. Third, almost one-third of the age-eligible women refused to participate in the study; however, we do not know if those women would have met the rest of the eligibility criteria for study enrollment or why these women refused. These women did not consent to participate; therefore, baseline characteristics of these women were not available to compare with study population. Fourth, potential hospital-related logistical challenges were neither ascertained nor studied, including procedure costs while in hospital, the potential impact on hospital length of stay, and availability of physicians, nurses, and other supporting staff needed for inpatient CRC screening. Fifth, this study also did not elicit the prevalence of contraindications associated with screening colonoscopy; admissions with acute colitis or diverticulitis, concern for bowel perforations, prior surgeries including anatomic issues, or difficulties related to anesthesia. Sixth, a large portion of women non-adherent to CRC screening who consented to the study are lost to follow-up after hospital discharge. We made up to 3 attempts via phone to reach out to these women and were unsuccessful. Finally, while this study found that most hospitalized women who were due for CRC screening would accept an inpatient colonoscopy, and while our anecdotal observations are that few hospitalized patients declined recommended medical tests, it is unclear how many of these patients would consent and undergo the procedure.

## Conclusion

A significant number of hospitalized women are non-adherent to CRC screening and value the opportunity to have CRC screening addressed during their stay. Inpatient hospital stay may be a feasible time to educate, counsel, and promote CRC screening to help ameliorate persistent and prevalent disparities in vulnerable hospitalized populations as well as to reduce the outpatient burden on primary care providers. Future studies are required to evaluate the feasibility of CRC screening while patients are hospitalized without extending hospital length of stay.

## Figures and Tables

**Table 1.** Characteristics Study Population

Characteristics^*^	All Study Population (n = 510)
Age, years, mean (SD)	60.5 (6.9)
Race	
Caucasians, n (%)	309 (61)
African American, n (%)	184 (36)
Others, n (%)	17 (3)
Married or living with a partner, n (%)	155 (31)
High school or more years of education, n (%)	406 (80)
Employment status, n (%)	113 (22)
Chronic disable, wheelchair or bed bound n (%)	25 (5)
Annual household income <$20 000, n (%)	225 (45)
Uninsured, n (%)	2 (0.4)
No primary care physician, n (%)	46 (9)
Presenting to hospital from home, n (%)	500 (98)
Admitted as observation, n (%)	29 (6)
Principle diagnosis at admission, n (%)	
General internal medicine, n (%)	161 (32)
Cardiovascular, n (%)	78 (15)
Pulmonary, n (%)	94 (18)
Gastrointestinal, n (%)	65 (13)
Neurology, n (%)	7 (1)
Nephrology, n (%)	34 (7)
Oncology, n (%)	8 (1)
Rheumatology, n (%)	18 (4)
Psychiatry, n (%)	4 (1)
Infectious disease, n (%)	25 (5)
Others, n (%)	16 (3)
Discharge from hospital to home, n (%)	496 (97)
Length of stay in days, mean (SD)	4.9 (5.2)
Non-adherent to colon cancer screening, n (%)	137 (27)
Never had screening colonoscopy, n (%)	173 (33)
High risk for colon cancer^†^, n (%)	45 (9)
Family history of colon cancer^Ŧ^, n (%)	58 (12)
Non-adherent to screening mammography, n (%)	169 (33)
5-year risk prediction using Gail model ≥1.7%^¤^, n (%)	210 (41)
Current Smoker, n (%)	162 (32)
Alcohol use, n (%)	132 (26)
Mean BMI, kg/m^2^, (SD)	33.2 (10.5)
Age-adjusted CCI >3^¥^, n (%)	309 (61)
Total comorbidities (excluding CCI)^◊^, mean (SD)	3.2 (1.9)

^*^For some patients, the variables had missing value;^ †^History of Lynch syndrome, familial adenomatous polyposis, or inflammatory bowel disease; ^Ŧ^Family history for a first-degree relative with colorectal cancer; ^¤^Gail score was estimated using the National Cancer Institute Breast Cancer Risk Tool (http://www.cancer.gov/bcrisktool/); ^¥^CCI scores of 0, 1, 2, and 3 predicting 10-year survival rates of 93%, 73%, 52%, and 45%, respectively; ^◊^Comorbidities excluded diseases accounted for CCI and included hypertension, heart disease, hypercholesterolemia, atrial fibrillation, history of pulmonary embolism or deep venous thrombosis, obstructive sleep apnea, osteoporosis, depression, chronic hepatitis, parkinsonism, hypothyroidism, nephrolithiasis, and anemia.

CCI, Charlson comorbidity index; SD standard deviation.

**Table 2.** Perspective About Colorectal Cancer and Screening in Hospitalized Women

Perspective^Ŧ^	All Study Population (n = 510)	Adherent (n = 373)	Non-adherent (n = 137)	*P* ^*^
Knowledge about colorectal cancer and screening				
Watched or heard advertisement encouraging to get colorectal cancer screening, n (%)	382 (77)	290 (80)	92 (68)	**.003**
Knew the age when a person with average risk for colorectal cancer starts screening, n (%)	202 (40)	144 (39)	58 (42)	.46
Knew the name of the test for colorectal cancer screening, n (%)	285 (56)	238 (64)	47 (34)	<.001
Perceived susceptibility				
It is likely that I will get colorectal cancer, agree/strongly agree, n (%)	89 (17)	67 (18)	22 (16)	.70
Colorectal cancer is fatal if not treated, agree/strongly agree, n (%)	487 (96)	360 (97)	127 (93)	.09
If I get a colonoscopy and nothing is found, I won’t worry as much about colorectal cancer, agree/strongly agree, n (%)	344 (67)	251 (67)	93 (68)	.90
I thought that colonoscopy would be painful, agree/strongly agree, n (%)	249 (49)	187 (50)	62 (45)	.33
Knew anyone who had screening colonoscopy, n (%)	373 (73)	286 (77)	87 (64)	**.004**
Encouraged by experience of anyone who had screening colonoscopy, agree/strongly agree, n (%)	166 (45)	140 (49)	26 (30)	**.002**
Perceived benefits				
Colorectal cancer can be detected at an early stage, agree/strongly agree, n (%)	488 (96)	357 (96)	131 (96)	1.00
Early detection of colorectal cancer improves the prognosis and chances for survival, agree/strongly agree, n (%)	497 (97)	366 (98)	131 (96)	.12
Perceived barriers				
Difficult to schedule colonoscopy screen, agree/strongly agree, n (%)	66 (13)	41 (11)	25 (18)	**.03**
I am afraid to have a colonoscopy because I don’t understand what will be done, agree/strongly agree, n (%)	90 (18)	47 (13)	43 (31)	**<.001**
Don’t know how to prepare for test, agree/strongly agree, n (%)	88 (17)	47 (13)	41 (30)	**<.001**
No transportation to get to the test, agree/strongly agree, n (%)	98 (19)	60 (17)	38 (28)	**.003**
Test is too expensive, agree/strongly agree, n (%)	82 (16)	55 (15)	27 (20)	.17
I have other problems more important than getting a colonoscopy, agree/strongly agree, n (%)	129 (25)	80 (21)	49 (36)	**.001**
Not counseled by my doctor about the need to do it, agree/strongly agree, n (%)	93 (18)	47 (13)	46 (34)	**<.001**
Unable to get a convenient appointment, agree/strongly agree, n (%)	48 (9)	29 (8)	19 (14)	**.04**
Having a colonoscopy exposes me to unnecessary harm, agree/strongly agree, n (%)	42 (8)	28 (8)	14 (10)	.36
I am afraid to have a colonoscopy because I might find out something is wrong, agree/strongly agree, n (%)	89 (18)	51 (14)	38 (28)	**<.001**

^Ŧ^For some patients, the variables had missing value; ^*^Chi-square test (Yates corrected *P* value where at least 20% of frequencies were <5). P ≤ .05 is significant.

**Table 3.** Care Received During Hospital Admission and Preferences for Screening

Questions^Ŧ^	All Study Population (n = 510)	Adherent (n = 373)	Non-adherent (n = 137)	*P* ^*^
Ever had screening colonoscopy, n (%)	337 (66)	317 (85)	20 (15)	**<.001**
Counseled about the importance of colorectal cancer screening by primary care provider, n (%)	345 (74)	277 (80)	68 (56)	**<.001**
Preference of screening colonoscopy location assuming can be done anywhere, n (%)				
Inpatient setting during hospitalization	254 (50)	193 (52)	61 (45)	**<.001**
Outpatient clinic colonoscopy	128 (25)	95 (26)	33 (24)	
Will not do either setting.	39 (8)	15 (4)	24 (18)	
Either setting will be fine	84 (17)	68 (18)	16 (12)	
Someone talked to me about colorectal cancer screening during this hospitalization, n (%)	32 (6)	28 (8)	4 (3)	.06
Study intervention – One-on-one bedside education with handout about colorectal cancer and counseling about importance of colorectal cancer screening				
I think it is important for healthcare providers to discuss colorectal cancer screening while patients are in the hospital, agree/strongly agree, n (%)	453 (89)	338 (91)	115 (84)	**.04**
During a hospitalization, I would agree to have an inpatient screening colonoscopy, if it was due and it was offered, agree/strongly agree, n (%)	399 (78)	309 (83)	90 (66)	**<.001**

^Ŧ^For some patients, the variables had missing value; ^*^Chi-square test (Yates corrected *P* value where at least 20% of frequencies were <5). P ≤ .05 is significant.
